# Integrative analysis of long noncoding RNAs dysregulation and synapse-associated ceRNA regulatory axes in autism

**DOI:** 10.1038/s41398-023-02662-5

**Published:** 2023-12-06

**Authors:** Miaomiao Jiang, Ziqi Wang, Tianlan Lu, Xianjing Li, Kang Yang, Liyang Zhao, Dai Zhang, Jun Li, Lifang Wang

**Affiliations:** 1 National Clinical Research Center for Mental Disorders (Peking University Sixth Hospital), NHC Key Laboratory of Mental Health (Peking University), Peking University Sixth Hospital, Peking University Institute of Mental Health, Beijing, China; 2 Beijing Key Laboratory of Mental Disorders, National Clinical Research Center for Mental Disorders & National Center for Mental Disorders, Beijing Anding Hospital, Capital Medical University, Beijing, China; 3 Advanced Innovation Center for Human Brain Protection, Capital Medical University, Beijing, China; 4 Guangdong Key Laboratory of Mental Health and Cognitive Science, Institute for Brain Research and Rehabilitation (IBRR), South China Normal University, Guangzhou, China

**Keywords:** Molecular neuroscience, Autism spectrum disorders

## Abstract

Autism spectrum disorder (ASD) is a complex disorder of neurodevelopment, the function of long noncoding RNA (lncRNA) in ASD remains essentially unknown. In the present study, gene networks were used to explore the ASD disease mechanisms integrating multiple data types (for example, RNA expression, whole-exome sequencing signals, weighted gene co-expression network analysis, and protein-protein interaction) and datasets (five human postmortem datasets). A total of 388 lncRNAs and five co-expression modules were found to be altered in ASD. The downregulated co-expression M4 module was significantly correlated with ASD, enriched with autism susceptibility genes and synaptic signaling. Integrating lncRNAs from the M4 module and microRNA (miRNA) dysregulation data from the literature identified competing endogenous RNA (ceRNA) network. We identified the downregulated mRNAs that interact with miRNAs by the miRTarBase, miRDB, and TargetScan databases. Our analysis reveals that *MIR600H*G was downregulated in multiple brain tissue datasets and was closely associated with 9 autism-susceptible miRNAs in the ceRNA network. *MIR600HG* and target mRNAs (*EPHA4*, *MOAP1*, *MAP3K9*, *STXBP1*, *PRKCE*, and *SCAMP5*) were downregulated in the peripheral blood by quantitative reverse transcription polymerase chain reaction analysis (false discovery rate <0.05). Subsequently, we assessed the role of lncRNA dysregulation in altered mRNA levels. Experimental verification showed that some synapse-associated mRNAs were downregulated after the *MIR600HG* knockdown. BrainSpan project showed that the expression patterns of *MIR600HG* (primate-specific lncRNA) and synapse-associated mRNA were similar in different human brain regions and at different stages of development. A combination of support vector machine and random forest machine learning algorithms retrieved the marker gene for ASD in the ceRNA network, and the area under the curve of the diagnostic nomogram was 0.851. In conclusion, dysregulation of *MIR600HG*, a novel specific lncRNA associated with ASD, is responsible for the ASD-associated miRNA-mRNA axes, thereby potentially regulating synaptogenesis.

## Introduction

Autism spectrum disorder (ASD) refers to a group of early-onset, lifelong, clinically heterogeneous, neurodevelopmental disorders with deficits in social functioning and the presence of repetitive and restricted behaviors or interests [1]. ASD also manifests significant genetic heterogeneity; thousands of common variants and rare, de novo single nucleotide mutations are estimated to contribute to ASD [1, 2]. Some studies have shown that ASD-associated mutations affect both coding and noncoding parts of the genome [3]. Most annotation sites in the human genome are noncoding, and a significant portion of noncoding transcripts are represented by long noncoding RNAs (lncRNAs) [4], which are defined as RNA molecules with >200 nucleotides. Among all other ncRNAs, lncRNAs are highly expressed in the human brain-specific regions of the neural tissues [5]. The lncRNAs are also involved in brain development and neurogenesis; thus several lncRNAs lead to defective neurogenesis and abnormal neural circuits after knockdown or aberrant alternative splicing [6, 7]. However, the contribution of the regulatory mechanisms of these lncRNAs with respect to ASD is yet to be elucidated.

Recent studies have identified the contribution of genetic, environmental, epigenetic, neuropathological, and immunological factors [8, 9]. LncRNAs play a role in various biological processes, including epigenetic regulation, chromatin remodeling, and the regulation of transcription and translation levels [10]. Several studies have shown that lncRNA is involved in many key biological functions and responds to environmental factors in the brain [3]. lncRNAs are fundamental regulators of transcription and can regulate susceptibility genes involved in psychiatric disorders and neurodevelopment [11–13]. The disruptions in lncRNAs, such as SHANK2-AS and BDNF-AS, can affect synapse, neuron function, and the development of autism [14]. Hitherto, only a small fraction of lncRNAs in the brain has been studied. For example, two primate-specific lncRNAs, LINC00693 and LINC00689, are upregulated in the ASD cortex [11]. The competing endogenous RNA (ceRNA) theory proposes crosstalks between ncRNAs and coding RNAs via microRNA recognition elements (MREs), which are microRNA (miRNA) complementary sequences [15]. However, the specific role of ceRNA networks in ASD has not been elucidated.

In the present study, weighted gene co-expression network analysis (WGCNA) and differential analysis were performed to screen the disease-associated lncRNAs, while integrating multiple data sources (such as five human postmortem datasets, GTEx, and Brainspan) to probe into ASD disease mechanisms using gene networks. Quantitative reverse transcription polymerase chain reaction (qRT-PCR) assays confirmed the dysregulation of lncRNAs and target genes at the peripheral circulation level. The in vitro experiments characterized MIR600HG for regulating the synapse-related genes through the ceRNA mechanism. Herein, our comprehensive bioinformatics analysis provides a framework for assessing the functional participation of lncRNAs in ASD.

## Materials and methods

### Data collection and differential expression analysis

The RNA-seq data of the prefrontal cortex from 34 autism patients in the GSE59288 and 38 normal samples in the GSE51264 dataset [16], obtained by sequencing on the Illumina HiSeq 2000 GPL11154 platform, was collected from the NCBI Gene Expression Omnibus (GEO) database. The microarrays and RNA-seq datasets, including GSE30573 (high coverage RNA-seq), GSE64018 (high coverage RNA-seq), GSE113834 (expression microarray), and GSE28521 (expression microarray), used for validation were integrated (Supplementary Table S1). First, the downloaded SRA file contained the sequencing reads for each sample, and its quality control was performed by FastQC (version 0.11.5). Then, the filtered reads were used to map to the hg38 genome reference genome (http://ftp.ensembl.org/pub/release-104/gtf/) using HISAT2 (version 2.1.0) [17] with default parameters. Third, the bam file was quantified using featureCounts [18] and filtered to remove the low-count genes expressed in <75% of samples. Finally, based on the raw counts matrix, we identified the differentially expressed protein-coding RNAs (DEmRNAs) and differentially expressed lncRNAs (DElncRNAs) using DEseq2 (|fold-change (FC)| > 1.3 and *p*-value < 0.05) for further analysis.

### Functional and pathway enrichment analyses

Gene ontology (GO), Kyoto Encyclopedia of Genes and Genomes (KEGG) pathway, gene set enrichment analysis (GSEA), and gene set variation analysis (GSVA) were performed using the R package “clusterProfiler” [19]. The cutoff criterion was *p* < 0.05. The datasets of multiple psychiatric disorders [20], the gene set of rare de novo variants associated with ASD by whole-exome sequencing study (WES) [21], different types of neuronal cell markers [22, 23], postsynaptic density [24], and embryonic development [21] were integrated for GSEA using Fisher’s exact test. The details of gene set selection and sources are summarized in Supplementary Table S2, *p*-values were adjusted for multiple comparisons using Benjamini–Hochberg correction to assess the false discovery rate (FDR).

### Screening of key modules based on WGCNA

Weighted gene co-expression network analysis facilitates the classification of genes based on their similar expression patterns. We used the “WGCNA” R package [25] to construct a scale-free co-expression network that adheres to the scale-free property. The scale-free network exhibits a power law distribution, which closely resembles the biological reality and demonstrates robustness. The soft-threshold power β = 6 was selected to construct a scale-free network. The adjacency matrix was transformed into a topological overlap matrix to describe the similarity between nodes. Furthermore, module-trait correlations were calculated to screen for modules with a significant correlation with autism (*p* < 0.05). The GSEA of each module was carried out using Fisher’s exact test. We extracted a protein-protein interaction (PPI) subnetwork for each associated module from the STRING database [26]. WGCNA was used to identify modules and susceptibility genes associated with autism. In this study, the corresponding gene significance (GS) and module membership (MM) of each gene in the core module were estimated. The genes satisfying the screening criteria (|MM| > 0.85 and |GS| > 0.2) were selected for further analysis. Pearson’s correlation coefficient was used to calculate the correlations between genes.

### Construction of ceRNA network

In order to identify candidate genes for the ceRNA network, the most significant lncRNAs and mRNAs based on WGCNA were intersected with the DElncRNAs and DEmRNAs. The differentially expressed candidate lncRNAs were input into the online database lncbaseV3 (https://diana.e-ce.uth.gr/lncbasev3) to identify the putative binding to miRNAs. We also identified the targeted miRNAs that interact with candidate mRNAs based on interactions generated by the miRTarBase [27], miRDB [28], and TargetScan [29] databases. Next, we searched for case-control studies exploring the differentially expressed miRNAs (DEmiRNAs) between patients with autism and healthy controls from multiple documents [30]. The interactions between DEmiRNAs and lncRNAs or mRNAs were integrated to construct a hub ceRNA regulatory network.

### Expression pattern analysis of ceRNA network

Pearson’s correlation analysis was used to determine any positive correlations between DEmRNAs and DElncRNAs. The tissue-expression heterogeneity of DEmRNAs and DElncRNAs in the ceRNA network was explored using the Genotype-Tissue Expression database (GTEx) (https://www.gtexportal.org/) and FUMA GWAS (https://fuma.ctglab.nl) [31]. Further, we retrieved spatial and temporal expression of the transcriptome in the human brain generated from the BrainSpan project (http://www.brainspan.org/).

### Participants

This study enrolled 70 Han Chinese autistic children (male/female ratio: 50/20) with a mean age of 3.68 (±0.61) (±standard deviation) years. A total of 75 age- and sex-matched healthy children (male/female ratio: 55/20) from the same ethnic group were selected as controls. All participants were recruited at the Peking University Sixth Hospital (Beijing, China). The peripheral blood samples were collected from all participants. Individuals with ASD were diagnosed based on DSM-IV criteria and had no other neuropsychiatric, metabolic, or immune-related conditions.

### Quantitative reverse transcription polymerase chain reaction

The RNA was extracted using the Tiangen RNAsimple Total RNA Kit (Tiangen, DP419, Beijing, China) and reverse-transcribed using a FastKing reverse transcriptase kit (Tiangen, KR116-02, Beijing, China), TransGen TransScript miRNA First-Strand cDNA Synthesis SuperMIX (TransGen, Beijing, China), and lnRcute lncRNA First Strand cDNA Synthesis Kit (Tiangen, KR202-02, Beijing, China). qRT-PCR was performed on a LightCycler 96 (Roche, Switzerland). The expression of the target lncRNA and mRNAs was normalized to that of the control *GAPDH*. For miRNAs, data were normalized with endogenous control *RNU6*. All primers are listed in Supplementary Table S3. For the robustness of the results, genes with low gene expression abundance (CT value > 32) were excluded. The relative quantitation for genes was calculated using the 2^−∆∆Ct^ method.

### Cell culture

The human embryonic kidney (HEK) 293 cells were obtained from the American Type Culture Collection (ATCC) and regularly checked for mycoplasma contamination. For cell culture, the cells were maintained in high-glucose Dulbecco’s modified Eagle medium (DMEM) containing 10% fetal bovine serum (FBS, GIBCO, 10099-141C, USA). For plasmid transfection, the cells were inoculated into 6-well plates and the plasmids were infected into 293 cells at 70% confluence. The expression plasmid [pLKO.1-CMV-copGFP-PURO or pLKO.1-sh-MIR600HG-CMV-copGFP-PURO] (Supplementary Table S3) was transfected using the jetPRIME® transfection reagent, following the manufacturer’s instructions. After 6 h, the medium was changed to fresh DMEM containing 10% FBS and cultured for 24 h.

### Diagnostic nomogram for ASD based on machine learning algorithms

A combination of support vector machine (SVM) recursive feature elimination (SVM-RFE) algorithm and random forest (RF) were used to screen the potential genes in the ceRNA network using the “e1071,” “randomForest” R package [32]. Next, a diagnostic nomogram was established based on the overlapping genes generated by SVM-RFE and RF algorithms. The nomograms were generated via the R package “rms”. Finally, the receiver operating characteristic (ROC) curve was used to investigate the efficiency of this diagnostic model. The area under the curve (AUC) > 0.65 was considered significant.

### Statistical analyses

All data were analyzed using SPSS. The differences in the relative expression for each mRNA and lncRNA between patients with autism and controls were examined using a nonparametric Mann–Whitney U test (two-tailed). Data are presented as the median and interquartile range. FDR was used for multiple comparison corrections. Downregulation of lncRNA and corresponding miRNA and mRNA regulatory axes was verified in cell experiments, and statistical significance was calculated using unpaired Student’s *t*-test (two-tailed). All data in accordance with the normal distribution were represented as means ± SEM. The threshold of significance accepted for all statistical analyses was the *p*-value or FDR < 0.05.

## Results

### DEmRNAs and DElncRNAs Identification

**Fig. 1:**
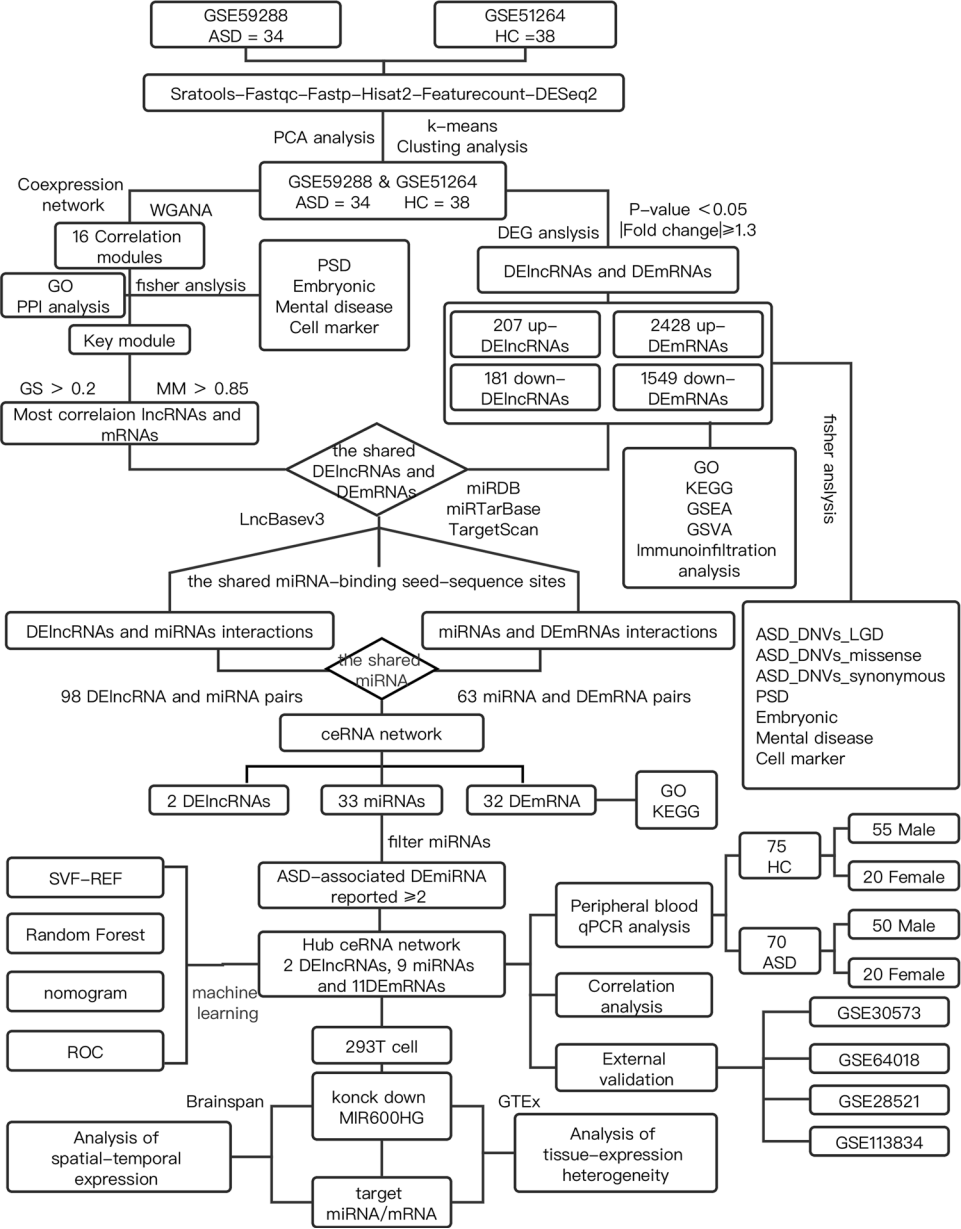
Flowchart of the overall approach. ASD autism spectrum disorder, DE differential expression, GO gene ontology, GSEA gene set enrichment analysis, GS gene significance, GSVA gene set variation analysis, HC healthy control, KEGG Kyoto Encyclopedia of Genes and Genomes pathway, LGD likely gene-disruptive mutations, MM module membership, PCA principal component analysis, PPI protein-protein interaction, PSD postsynaptic density, ROC receiver operating characteristic, SVM-REF support vector machine recursive feature elimination, WGCNA weighted gene co-expression network analysis.

**Fig. 2:**
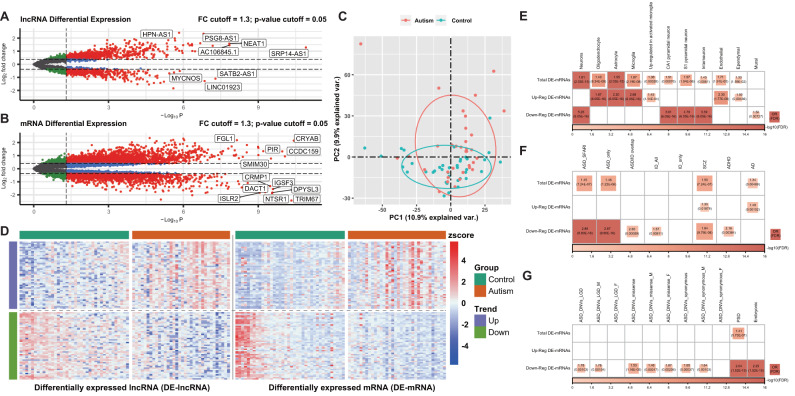
Differential gene expression analysis between ASD and CTL samples and enrichment analysis (Fisher’s exact test). **A** Volcano plot for DElncRNAs. **B** Volcano plot for DEmRNAs. Differentially expressed (DE) mRNAs and lncRNAs are highlighted in red. **C** PCA analysis. **D** Heatmap of DEmRNAs and DElncRNAs. **E** Heatmap showing enrichment of markers for different types of neural cells. **F** Heatmap showing enrichment of ASD risk genes from *SFARI* (ASD SFARI), intellectual disability genes (ID all), schizophrenia genes (SCZ), attention deficit hyperactivity disorder genes (ADHD), and Alzheimer’s disease genes (AD). “ASD/ID overlap,” the overlap between the “ASD SFARI” and “ID all” sets. “ASD only” and “ID only,” non-overlapping ASD SFARI and ID genes, respectively. **G** Heatmap shows the enrichment of genes affected by de novo variants (DNVs), including likely gene-disrupting (LGD), missense, synonymous in ASD (M, male; F, female), and genes encoding proteins in the postsynaptic density (PSD), genes expressed preferentially in human embryonic brains (Embryonic). Fisher’s exact test (two-tailed) with FDR correction was applied for enrichment. In (**E**–**G**), enrichment odds ratios (OR) and FDR-corrected *p*-values are shown for enrichment with FDR < 0.05. PCA principal component analysis, FC fold-change.

We profiled differential gene expression (DGE) analysis in 72 postmortem brain tissue samples from 34 ASD cases (GSE59288) and 38 controls (CTL) (GSE51264). A filtering flowchart for the study is shown in Fig. 1. The 388 DElncRNAs (207 upregulated and 181 downregulated) were identified between CTL and ASD samples. We also identified 2428 up- and 1549 downregulated DEmRNAs (Fig. 2 and Supplementary Table S4). Principal component analysis (PCA) revealed the following findings (Fig. 2C): the ASD samples were separated from CTL samples by PCA1.

### Enrichment analysis (Fisher’s exact test) for gene sets of interest

To explore the correlation between the DEmRNAs and the neural cell type marker gene sets, multiple psychiatric disorders, and broad scope of ASD risk genes, including genes with evidence of de novo risk variants, we systematically assessed whether the DEmRNAs are enriched for these genes (Fisher’s exact test, FDR < 0.05; “Methods” section). We observed that the DEmRNAs are significantly enrichment for a variety of neural cell type marker genes. The ASD-upregulated genes are associated with oligodendrocyte, astrocyte and microglial function, while the downregulated genes are associated with neurons, CA1 pyramidal neurons, S1 pyramidal neurons, interneurons and mural cells (Fig. 2E and Supplementary Table S2). The other types of immune cell marker analysis found that CD4+ memory T cells were enriched in the upregulated genes (Supplementary Fig. S1). Interestingly, the downregulated DEmRNAs are enriched for genes causally connected with autism (Fig. 2F) but not for other brain disease-associated genes. We also observed enrichment for the downregulated DEmRNAs hit by de novo variations (likely gene-disruptive (LGD) mutations, missense, and synonymous). These downregulated DEmRNAs also significantly overlapped with encoding postsynaptic density (PSD) proteins and embryonically expressed genes (Fig. 2G and Supplementary Table S2).

### Pathway enrichment analyses

**Fig. 3:**
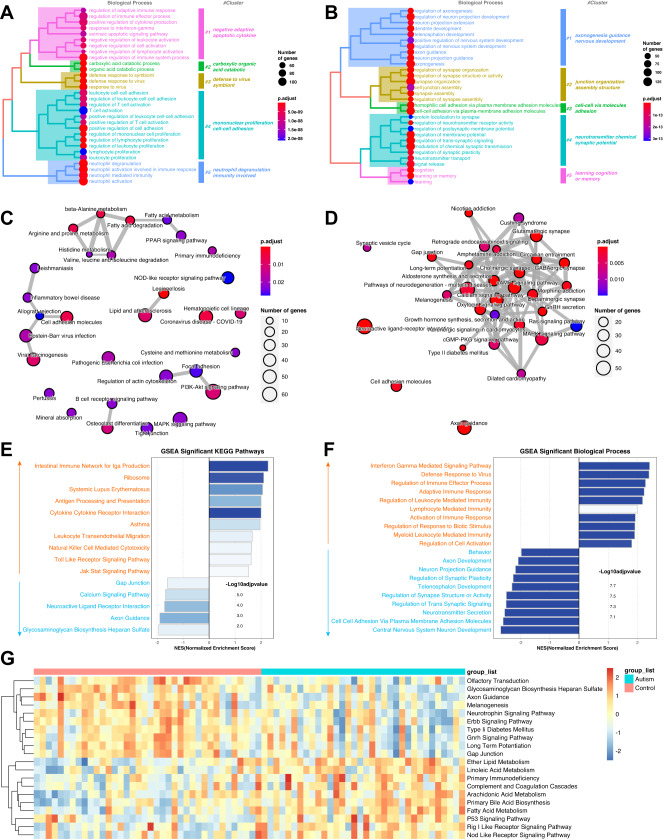
Integrated enrichment analysis. **A** Heatmap plot of BP enriched terms for the upregulated DEmRNAs. **B** Heatmap plot of BP enriched terms for the downregulated DEmRNAs. **C**, **D** Enrichment map of KEGG analysis for the up- and downregulated DEmRNAs. **E**, **F** Significant KEGG pathway and BP terms by GSEA analysis. **G** Significant KEGG pathway by GSVA analysis. BP biological process, KEGG Kyoto Encyclopedia of Genes and Genomes pathway, GSEA gene set enrichment analysis, GSVA gene set variation analysis.

The hierarchical clustering of biological process (BP) terms by measuring similarity revealed that the upregulated set was related to immunity and cell adhesion function (Fig. 3A). The downregulated set was divided into several categories, including “regulation of axonogenesis,” “nervous development,” and “synaptic function” (Fig. 3B). Next, we performed a series of pathway enrichment analyses to characterize these genes. The NOD-like receptor signaling pathway and the PI3K-Akt signaling pathway are two immune-related processes that are linked to the upregulated DEmRNAs (Fig. 3C). Notably, the downregulated groups are involved in the activation of synaptic-related pathways (such as GABAergic synapses and glutamatergic synapses), Calcium signaling pathways, and oxytocin signaling pathways; these pathway targets form a network of tighter interactions than accidentally expected, providing independent confirmation of pathway-level co-regulation (Fig. 3D).

Based on these findings, we defined the changing trend of molecular pathways and conducted GSEA analysis on the two groups of samples (Fig. 3E, F). The results of the GSEA analysis on samples shared similarities with those of GO and KEGG analyses of the DEGs and also exhibited specific characteristics. Interestingly, the intestinal immune network for the IgA production pathway is upregulated in ASD (Fig. 3E). The details of GSEA pathways are described in Supplementary Fig. S2. The results of the GSVA analysis (Fig. 3G) further confirmed the reliability of the GSEA results. First, the pathways related to the inflammation immune microenvironment-related pathways (RIG-I-like receptor signaling pathway and NOD-like receptor signaling pathway) were significantly upregulated in the ASD group, as well as the metabolic pathways of fatty acids. In line with the desired results, the downregulated pathways in the ASD group were largely consistent with the results of GSEA, which mainly focused on the pathways related to axon development, neuron projection guidance, and regulation of synapse structure or activity (Fig. 3F, G).

### Perturbation of lncRNA co-expression modules in ASD brain

**Fig. 4:**
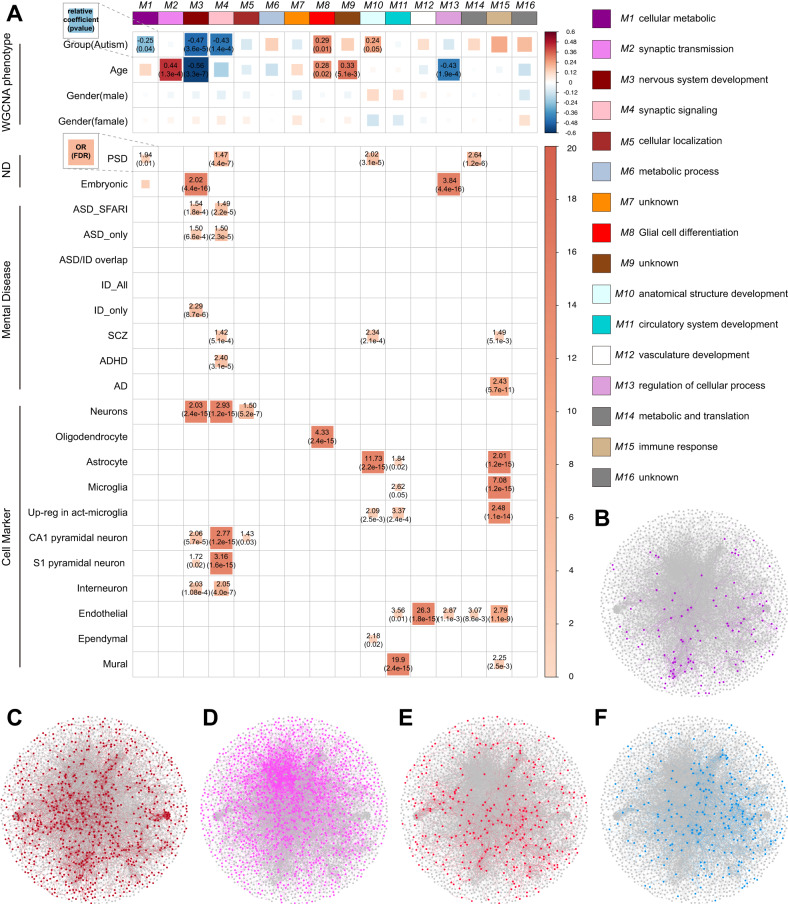
lncRNA and mRNA co-expression modules dysregulated in postmortem ASD cortex. **A** Pearson’s correlation analysis between module eigengenes and different covariates (upper part). Correlation coefficients and *p*-values are shown at *p* < 0.05. The right side is named according to the BP of each module. The module enrichment analysis (Fisher’s exact test, FDR < 0.05) is shown on the lower part. Enrichment OR and FDR-corrected *p-*values are shown for enrichment with FDR < 0.05. **B**–**F** PPI network construction for five modules (M1, M3, M4, M8, and M10) was correlated with the disease status. ND neurodevelopment, BP biological process, PPI protein-protein interaction.

To further assess the correlations between lncRNA expression changes and disease status, we applied WGCNA (Supplementary Fig. S3 and Supplementary Table S5) to assign the lncRNAs and mRNAs to co-expression modules. Subsequently, 16 modules (Fig. 4A) were identified, and their biological function was examined. Next, we identified five modules that were correlated with the disease status (Pearson’s correlation, *p* < 0.05); three downregulated (M1, M3, and M4) and two upregulated (M8 and M10) in ASD samples (Supplementary Fig. S3F–J). The GSEA (Fisher’s exact test) of each module (Supplementary Table S6) showed that the upregulated M8 module was associated with glial cell differentiation and enriched in oligodendrocytes. On the other hand, the M3 (nervous system development) and M4 (synaptic signaling) modules showed highly significant enrichment for known autism susceptibility genes and multi-neuronal markers. The M3 modules showed significant correlations with age and hence, were excluded from subsequent analysis. However, M4 was significantly correlated with disease status but not correlated with critical experimental covariates (age and sex) (Fig. 4A). Next, we plotted the PPI network of the modules to show functional interactions between proteins, with the strongest protein interactions for the M4 module (Fig. 4B–F). The key gene co-expression module (M4) was significantly correlated with ASD, enriched for DEGs between ASD and control (Supplementary Table S4). The screening criteria for the DElncRNAs and DEmRNAs of the M4 module were based on the MM and GS assessment (Supplementary Table S7; “Methods”).

### Identification of the ceRNA network associated with ASD

**Fig. 5:**
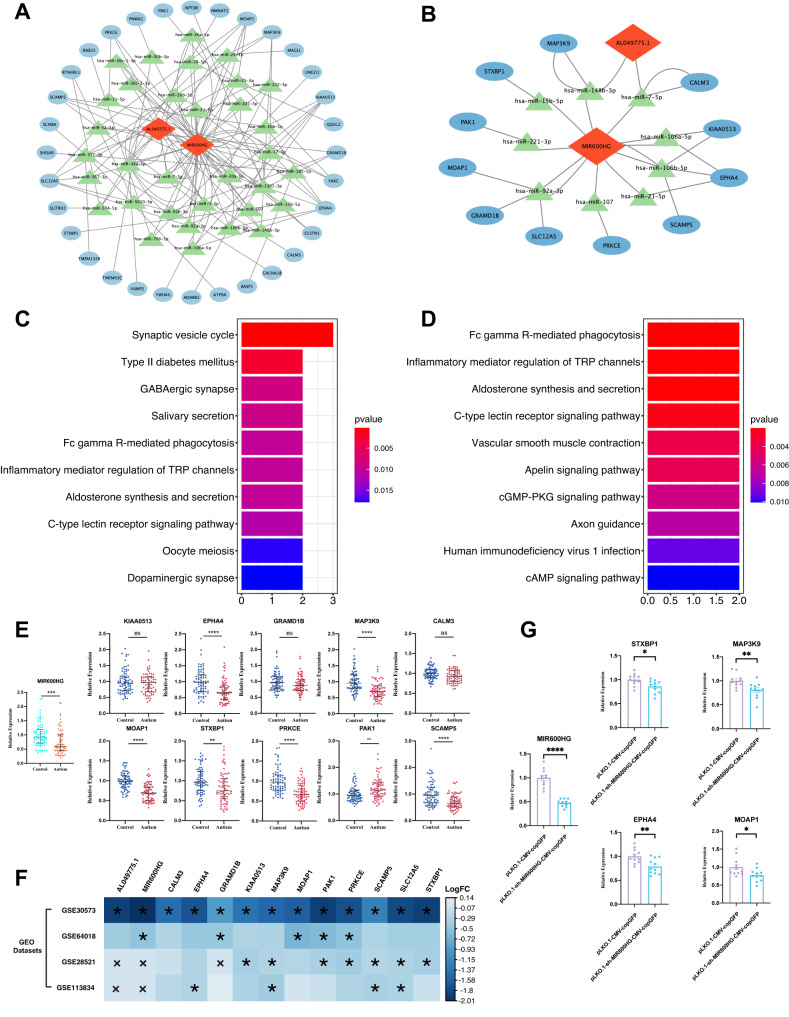
ceRNA network, functional enrichment analysis, and validation of the ceRNA network. **A** The preliminary lncRNA-miRNA-mRNA (ceRNA) regulatory networks. **B** Key ceRNA network. **C**, **D** KEGG enrichment analysis of preliminary or key ceRNA network. **E** Expression of 10 hub DEmRNAs and 1 hub DElncRNA in patients with autism (*n* = 70) and healthy controls (*n* = 75). Mann–Whitney U test was used for statistical analysis, and FDR was used for multiple testing corrections with each dot representing an individual. Data are presented as the median and interquartile range. * FDR < 0.05, ** FDR < 0.01, *** FDR < 0.001, **** FDR < 0.0001. **F** Validation of the expression of ceRNA network marker genes in four datasets. * |FC| > 1.3 and *p*-value < 0.05; x, the chip data cannot detect mRNA and lncRNA in the sample. **G** Experimental validation of a downregulated lncRNA (MIR600HG) and the corresponding mRNA regulatory axis. Data are presented as individual data points, with bar plots showing the mean and standard deviation (*n* = 11 for each group). Statistical significance was calculated by Student’s *t*-test, * *P* < 0.05, ** *P* < 0.01, **** *P* < 0.0001, Mean ± SEM.

We first searched for the target miRNAs of the DElncRNAs from the M4 modules and identified 98 lncRNA-miRNA interactions, as described above. Next, the potential interactions of 63 miRNAs with DEmRNA, based on a collection of interactions supported simultaneously by three miRNA target prediction databases, were identified. A preliminary ceRNA regulatory network exhibited a positive correlation between the expression of lncRNA and mRNA (Fig. 5A; Supplementary Table S8). Next, we searched for case-control studies on the miRNA dysregulation between patients with autism and healthy controls from multiple documents. A total of 74 ASD-associated DEmiRNAs, reported from at least two previous case-control studies (Supplementary Table S9), were selected. The lncRNA-miRNA-mRNA associations with the DEmiRNA as the core were screened, and a hub ceRNA network consisting of 2 DElncRNAs, 9 DEmiRNAs, and 11 DEmRNAs was constructed (Fig. 5B; Supplementary Table S8). Further enrichment analyses revealed the ceRNA network-related signaling pathways, including “synaptic vesicle cycle,” “Fc gamma R-mediated phagocytosis,” and “positive regulation of JUN kinase activity” (Fig. 5C, D; Supplementary Table S10). The GO enrichment analysis of key ceRNA networks revealed that syntaxin-binding protein 1 (*STXBP1*) and secretory carrier membrane protein 5 (*SCAMP5*) are involved in the positive regulation of calcium-dependent exocytosis. And tyrosine kinase ephrin receptor A4 (*EPHA4*), protein kinase C epsilon (*PRKCE*), and *STXBP1* are jointly involved in the regulation of transsynaptic signaling (Supplementary Table S10).

### Validation of ceRNA networks

We assessed the role of lncRNA dysregulation in altered mRNA levels. In the ceRNA network, *MIR600HG* and *AL049775.1* had strong correlations with 11 DEmRNAs, as assessed by Pearson’s correlation analysis (correlation coefficients > 0.75) (Supplementary Fig. S4; Supplementary Table S11). Of these, *MIR600HG* and *MAP3K9* had the strongest correlation with high correlation coefficients at 0.91. Furthermore, *STXBP1* has a positive correlation with another mRNA (correlation coefficients > 0.85). For example, *STXBP1* had interacts with *MAP3KP9* (r = 0.93), *SCAMP5* (r = 0.94), *PRKCE* (r = 0.94), *EPHA4* (r = 0.86), and *MOAP1* (r = 0.94). In addition, four microarrays and RNA-seq datasets used for validation were integrated, and the downregulated expression of marker genes from ceRNA networks was observed in the autistic brain tissue (Fig. 5F). *MIR600HG* was found to be downregulated in GSE30573 (log2FC = −2.01) and GSE64018 (log2FC = −0.49) (Fig. 5F and Supplementary Table S12).

### Validation of the hub genes in clinical samples

*MIR600HG* is the most closely related to nine known autism-susceptible miRNAs (Fig. 5B). Among the identified lncRNA-miRNA-mRNA interactions, we selected a DE-lncRNA (*MIR600HG*, which was downregulated in multiple datasets) for the following experimental validations. *MIR600HG* exhibited significant differential expression between patients with autism and controls by qRT-PCR (log2FC = −0.62, FDR = 2.44E-04) (Fig. 5E and Supplementary Table S13), and differences were observed in the male samples (log2FC = −0.64, FDR = 1.81E-03) instead of the females (Supplementary Fig. S5). *PAK1* was downregulated in the postmortem cortex and upregulated in whole blood. However, 6/10 DE-mRNAs (*SLC12A5* excluded) were significantly downregulated, including *EPHA4* (log2FC = −0.56, FDR = 1.45E-05), *MAP3K9* (log2FC = −0.47, FDR = 7.81E-07), *MOAP1* (log2FC = −0.51, FDR = 1.14E-10), *STXBP1* (log2FC = −0.38, FDR = 2.28E-03), *PRKCE* (log2FC = −0.54, FDR = 1.95E-08), and *SCAMP5* (log2FC = −0.54, FDR = 5.06E-05). The relative expressions of these mRNAs, except for *SCAMP5*, were significantly downregulated in the peripheral blood of both male and female patients (Supplementary Table S13; Supplementary Fig. S5). Concurrently, the prefrontal cortex samples obtained from male patients exhibited a significant downregulation in the expression levels of *MIR600HG* and its associated target genes. In contrast, the female samples displayed a declining trend, but the statistical analysis did not yield significant evidence to support this observation (Supplementary Fig. S6).

### Correlation verification of mRNA regulation by MIR600HG

We found that significant DE-lncRNA (*MIR600HG*) and six differential mRNAs were downregulated in the brain tissue and peripheral blood. We observed an upregulation trend in nine specific miRNAs following interference with *MIR600HG*. Notably, hsa-miR-148b-3p, hsa-miR-7-5p, hsa-miR-106b-5p, and hsa-miR-21-5p were significantly upregulated with statistical significance (Supplementary Fig. S7). Previous studies have reported upregulation of these miRNAs in the brain tissue of individuals with ASD (Supplementary Table S9). These findings provide further support for the regulatory role of *MIR600HG* in modulating the expression of these specific miRNAs. The results showed that *MIR600HG* knockdown affects the expression of its corresponding mRNAs (*STXBP1*, *MAP3K9*, *EPHA4*, and *MOAP1*) (Fig. 5G). Notably, we examined the expression of *MIR600HG* and the mRNA counterparts in various human tissues by GTEx profiles and found that these genes were enriched in the brain (Supplementary Fig. S8A). RNA-seq data from the BrainSpan project showed that *MIR600HG* is broadly expressed in brain regions, but developmentally regulated in the human brain from pregnancy to adulthood. In this study, six main brain regions (dorsolateral prefrontal cortex (DFC); ventrolateral prefrontal cortex (VFC); medial prefrontal cortex (MFC); orbital frontal cortex (OFC); hippocampus (HIP), and inferolateral temporal cortex (ITC)) were investigated. *MIR600HG* has spatiotemporal co-expression with the targeted mRNA at each developmental stage (prenatal, postnatal, and adulthood), which may have similar molecular pathways or functions. The expression patterns of *MIR600HG* and mRNA were similar in different human brain regions and at different stages of development (Supplementary Fig. S8B–G). Together, these findings hinted at the role of a new lncRNA *MIR600HG* in regulating ASD-associated miRNAs and mRNAs, suggesting a critical role of the ceRNA network in ASD.

### Construction of a diagnostic model based on machine learning algorithms

Machine learning algorithms were selected for the diagnostic modeling of the marker genes of ASD in the ceRNA network. First, SVM-RFE analysis revealed that the first 10 genes were identified as potential genes based on an optimum error rate (0.316). Similarly, the RF algorithm screened 11 ASD-associated diagnostic marker genes (Supplementary Fig. S9A). Finally, the common genes obtained by the above two algorithms, *PRKCE*, *CALM3*, *STXBP1*, *GRAMD1B*, *MAP3K9*, *PAK1*, *MOAP1*, *KIAA0513*, and *MIR600HG*, were utilized to construct a diagnostic nomogram (Supplementary Fig. S9B). The analyses of the AUC revealed that these 9 genes hold potential as diagnostic biomarkers. The AUC of the diagnostic nomogram was 0.851 for prefrontal cortex samples, and the diagnostic performance of the model was satisfactory (AUC > 0.65) in peripheral blood samples (Supplementary Fig. S9C–E).

## Discussion

The lncRNA and mRNA co-expression module was obtained by WGCNA analysis and detected along with the DE genes for the enrichment of autism-related gene signals. The ceRNA network was constructed by integrating the candidate miRNA gene sets from the literature. RT-PCR assays confirmed that lncRNAs and target genes are dysregulated at the peripheral circulatory level. Next, we hypothesized that *MIR600HG* regulating transcription-level genes through the mechanism of ceRNA may lead to ASD susceptibility. This model is supported by a positive correlation between the expression of lncRNAs and mRNAs affected by ASD; also, the regulation of miRNA and mRNA targets was assessed by lncRNA knockdown in cells. Overall, our findings suggested that ASD-associated transcriptomic changes may be partially attributed to lncRNA dysregulation.

*MIR600HG* is a common intersection of DGE analysis and co-expression M4 modules. WGCNA analysis determined that M4 modules (downregulated) are correlated with ASD and significantly overlapped with PSD proteins and synaptic signaling function. Furthermore, the function of upregulated genes and modules was related to immunity, as described previously [33, 34]. However, the upregulated genes did not show an enrichment of the genetic components. Previous studies indicated that lncRNA is a transcriptional and post-transcriptional regulator, and dysregulation of ncRNAs plays a critical role in the pathogenesis of ASD [6, 7, 11, 13]. *MIR600HG* in Alzheimer’s disease regulates Aβ accumulation [35] and is associated with tumors [36, 37]. In the present study, *MIR600HG* was downregulated in multiple datasets of postmortem brain tissue.

The dysregulation of *MIR600HG* expression and the role of the ceRNA regulatory axis in ASD have never been reported. Also, we highlighted that *MIR600HG* interacts with multiple mRNAs through autism-susceptible miRNAs (for example, hsa-miR-106a/b-5p, hsa-miR-107, hsa-miR-92a-3p, hsa-miR-15b-5p, hsa-miR-21-5p, and hsa-miR-148b-3p). The miRNA of interest has been associated with ASD, providing a useful set of upstream regulatory (lncRNA-miRNA axis) target genes. In this study, it was found that the expression of some miRNAs was significantly upregulated after inhibiting the expression of *MIR600HG* using shRNA knockdown. These findings further support the role of lncRNA as endogenous “sponges” for miRNAs [15]. These dysregulated miRNAs might affect the expression of genes related to autism and neurodevelopment [38–41]. The target mRNAs were downregulated in the brain tissue (GEO datasets) and peripheral blood (Han Chinese populations), including *STXBP1*, *EPHA4*, *MAP3K9*, *MOAP1*, *PRKCE*, and *SCAMP5*.

*STXBP1*, localized primarily in the cell body and axon, is associated with vesicle fusion and neurotransmitter release throughout development [42–44]. The inactivation of Munc18-1 in mice leads to widespread neurodegeneration [43] and synaptic impairments [45]. A decrease in STXBP1 by patient-derived induced pluripotent stem cells (iPSCs) to generate induced neurons resulted in neurite extension defects [46]. The mutations in *STXBP1* are associated with neurodevelopmental disorders, such as ASD, developmental disorders, and epileptic encephalopathy [47–49]. Recurrent de novo and likely gene-disruptive mutations for *STXBP1* have been reported in a Chinese ASD patient cohort [50].

*SCAMP5* is highly expressed in the brain and is involved in transmitting nerve signals, regulating axonal trafficking, synaptic localization, and synaptic plasticity [51]. De novo *SCAMP5* mutation causes a neurodevelopmental disorder with autistic features [52]. SCAMP5 has been shown to interact with soluble N-ethylmaleimide sensitive factor attachment protein receptors (SNAREs) molecules, which are important for intracellular vesicular trafficking and developmental psychiatry [53, 54]. *EPHA4* belongs to the A subgroup of Eph receptors, which are key players in synaptic plasticity and neural development [55, 56]. Rare changes in EPHA4/p.P775L are linked to ASD [48] and *EPHA3* identified as candidate genes in ASD [57]. Both EphA4 and EphA7 affect cortical neuronal migration during mouse brain development [56]. EphA4 knock-out (-/-) mice displayed impaired movements, which are associated with disruption of axon-guided function [58].

*MAP3K9* regulates signaling by the mitogen-activated protein kinase (MAPK) and c-Jun amino-terminal kinase (JNK) pathways [59]. The MAPK signaling pathway, critical for neurodevelopment, is involved in neurogenesis migration and the development of dendritic trees and spines [60]. Some studies have shown that the MAPK signaling pathway and mitochondrial dysfunction are involved in the pathogenesis of ASD [61]. Furthermore, in the present study, among the ceRNA network, *MIR600HG* had a strong correlation with DEmRNAs.

LncRNAs regulate gene expression in multiple ways at the epigenetic, chromatin remodeling, transcription, and translation levels, and as ceRNAs that attenuate the role of miRNAs on targeted messenger RNA (mRNA) involved in the development of tumors and neurological diseases [5, 10, 15, 62, 63]. Recently, the mechanisms of immune-related ceRNA regulation in ASD diseases have been deduced [64, 65]. However, the role of the synaptic-associated ceRNA regulatory axis in ASD has never been reported and remains largely unknown.

In the present study, we provided the corresponding ASD-associated ceRNA network and characterized the targets of *MIR600HG*-downregulated mRNA axis (for example, hsa-miR-21-5p/hsa-miR-106b-5p-*EPHA4* and hsa-miR-148b-3p-*MAP3K9* axis) in vitro. Thus, elucidating specific spatial and temporal expression patterns of lncRNAs is crucial to identifying the role of lncRNAs in nervous system development [66]. In this study, the expression patterns of MIR600HG and targeted mRNA were similar in different human brain regions and at different stages of development, suggesting that they may have similar molecular pathways or functions. Another study reported that the JNK and MAP kinase pathways play a crucial role in synaptic formation [67]. Interestingly, functional analysis revealed that *EPHA4* and *MAP3K9* genes are related to JUN kinase activation, and *EPHA4* and *STXBP1* are enriched in synaptic signal transduction and regulation of calcium ion-dependent exocytosis; all these are involved in the pathogenesis of ASD. Moreover, lncRNAs, such as metastasis-associated lung adenocarcinoma transcript 1 *(MALAT1*) [68], *BDNF-AS* [69], brain cytoplasmic RNA 1 (*BCYRN1*) [70] regulate synapsis and synaptic plasticity through alternative splicing or the expression of synaptic-related genes. In summary, *MIR600HG* potentially regulates the expression of synaptic function-related genes at the transcription level through ceRNA mechanisms.

Nevertheless, the present study has several limitations. For example, further functional exploration of lncRNAs and mRNAs with spatiotemporal expression pattern consistency is essential. Due to the species-specific expression of this particular lncRNA (*MIR600HG*) in humans, future investigations should prioritize the inclusion of appropriate animal models to validate and extend the findings of this study. The young brain samples from patients and patient-derived brain organoids that mimic early brain development will help in characterizing the dysregulation of lncRNAs and potential ceRNA regulation mechanisms in autism. In the future, the investigation of the manipulation of *MIR600HG* expression in the brain and studying its impact at the molecular and behavioral levels would provide a more comprehensive exploration of the functional significance of this lncRNA in brain development and behavior. Moreover, the present study focuses on transcriptomics because the transcription process is affected by many factors, and mRNA changes may vary between the blood and the brain. Thus, the current findings need to be replicated with larger samples and different ethnic backgrounds. The limited sample size of female ASD patients in our study may have contributed to the lack of statistical significance in the observed declining trend of *MIR600H*G expression and its associated target genes. Future studies with larger cohorts of female ASD patients are required to validate and further explore these observations.

In conclusion, dysregulation of MIR600HG potentially regulates the expression of synaptic function-related genes at the transcription level through ceRNA mechanisms. Consequently, our findings corroborate the role of lncRNA dysregulation and synaptic signaling-related ceRNA regulatory axis in ASD. Our comprehensive bioinformatics analysis provides a framework for assessing the functional participation of lncRNAs in ASD. The role of lncRNA in the development and function of the central nervous system needs to be investigated further.

### Supplementary information

Supplementary Figures

Supplementary Tables

## Data Availability

The original contributions presented in the study are included in the article/Supplementary Material, further inquiries can be directed to the corresponding authors.
