# Supplementary material for: Integrative analysis of long noncoding RNAs dysregulation and synapse-associated ceRNA regulatory axes in autism
Source: Transl Psychiatry. 2023 Dec 6;13:375. doi: 10.1038/s41398-023-02662-5 (PMC10700319; doi:10.1038/s41398-023-02662-5)
Supplement: Supplementary file 1 — Supplementary Figures [file 41398_2023_2662_MOESM1_ESM.docx]

***Supplementary Figures***

**Supplementary Fig. S1**. Multiple cell types in ASD brain tissue were assessed using Xcell.

**Supplementary Fig. S2**. Gene set enrichment analysis (GSEA).

**Supplementary Fig. S3**. Weighted gene coexpression network analysis (WGCNA).

**Supplementary Fig. S4**. Correlation analysis of ceRNA network in GSE59288 and GSE51264.

**Supplementary Fig. S5**. Validation of the ceRNA network marker genes in clinical (peripheral blood) samples.

**Supplementary Fig. S6**. The ceRNA network marker genes in GSE59288 (autism patients: n = 34) and GSE51264 (normal samples: n = 38) of prefrontal cortex samples.

**Supplementary Fig. S7**. Experimental validation of the miRNA expression associated with MIR600HG following shRNA knockdown of this lncRNA.

**Supplementary Fig. S8**. Gene expression heatmap.

**Supplementary Fig. S9**. Machine learning algorithms were applied for diagnostic modeling.

***
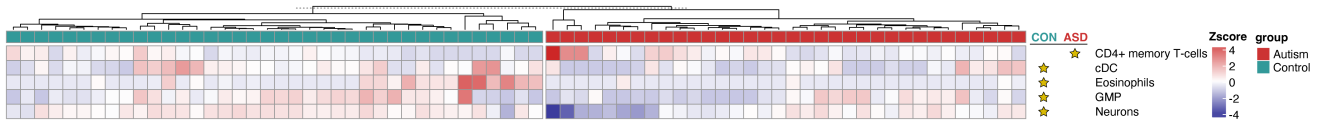
***

**Supplementary Fig. S1**. Multiple cell types in ASD brain tissue were assessed using Xcell. (<https://xcell.ucsf.edu/>). DC, dendritic cells；GMP, granulocyte-macrophage progenitor.


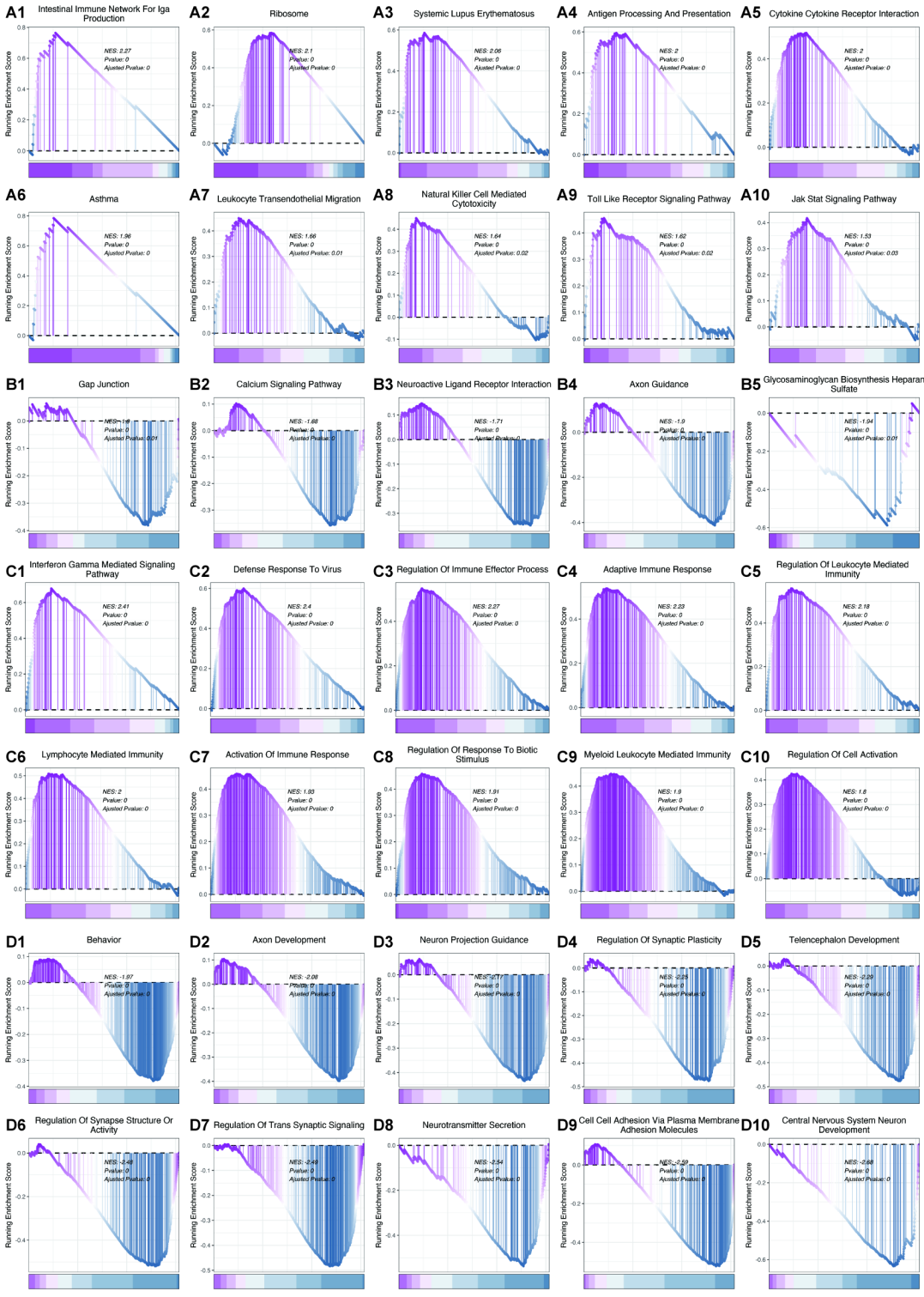


**Supplementary Fig. S2**. Gene set enrichment analysis (GSEA). (A1–A10) Mainly upregulated KEGG pathway based on GSEA. (B1–B5) Mainly downregulated KEGG pathway based on GSEA. (C1–C10) Mainly upregulated BP based on GSEA. (D1–D10) Mainly downregulated BP based on GSEA. BP, biological process; KEGG, Kyoto Encyclopedia of Genes and Genomes pathway.


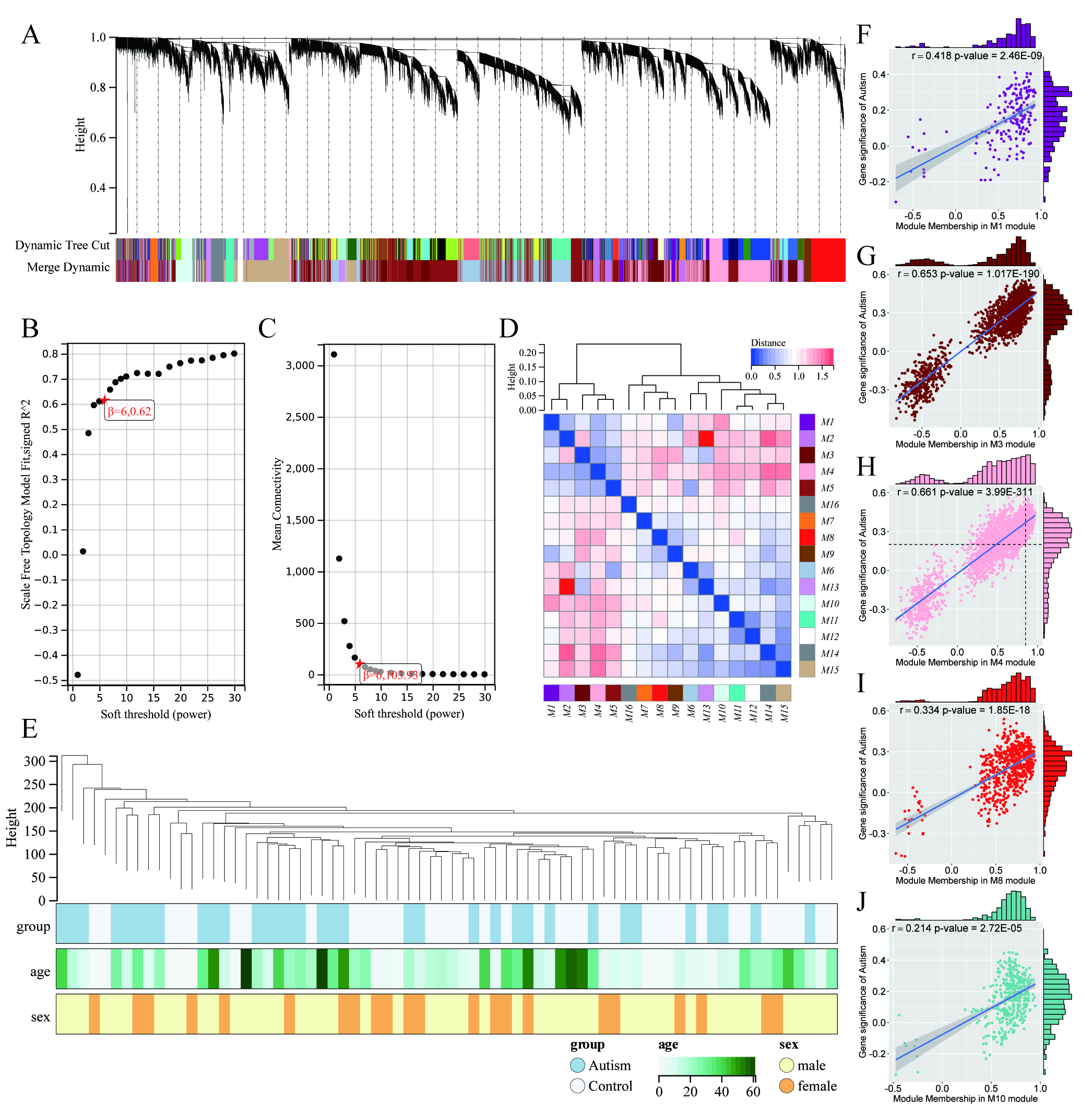


**Supplementary Fig. S3**. Weighted gene coexpression network analysis (WGCNA). (A) Clustering dendrogram. (B, C) Analysis of the scale-free fit index and mean connectivity through scale-free network construction. (D) Heatmap of module-trait correlations. (E) Hierarchical clustering of all samples. Information on diagnosis, age, and sex is indicated with color bars below the dendrogram. (F–J) Five modules related to autism. Dots are colored according to the module.

**
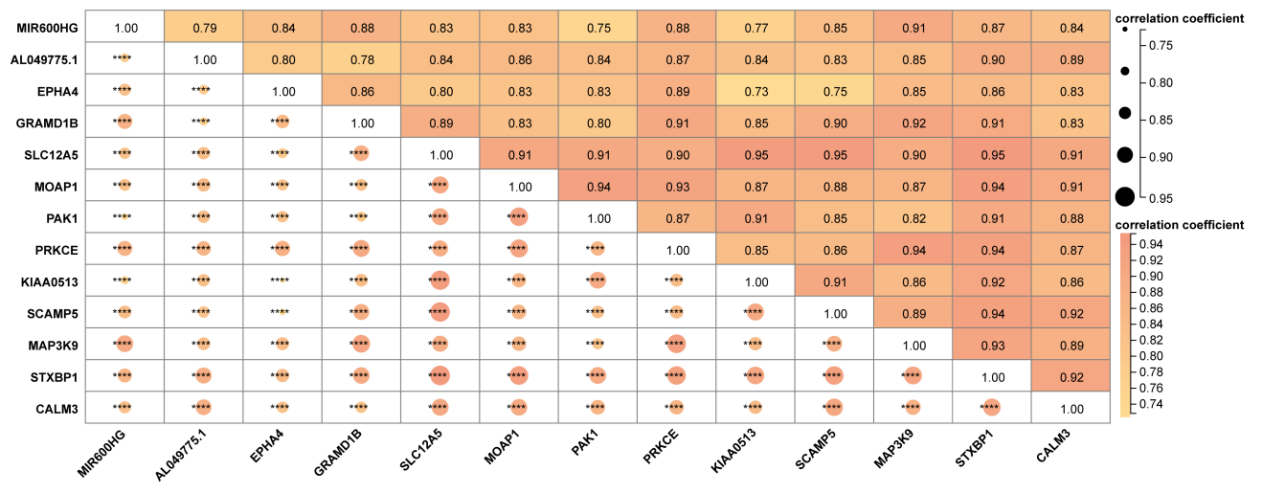
**

**Supplementary Fig. S4**. Correlation analysis of ceRNA network in GSE59288 and GSE51264. A positive correlation was detected between lncRNAs and target mRNAs. Pearson’s correlation coefficients and p-values are indicated.


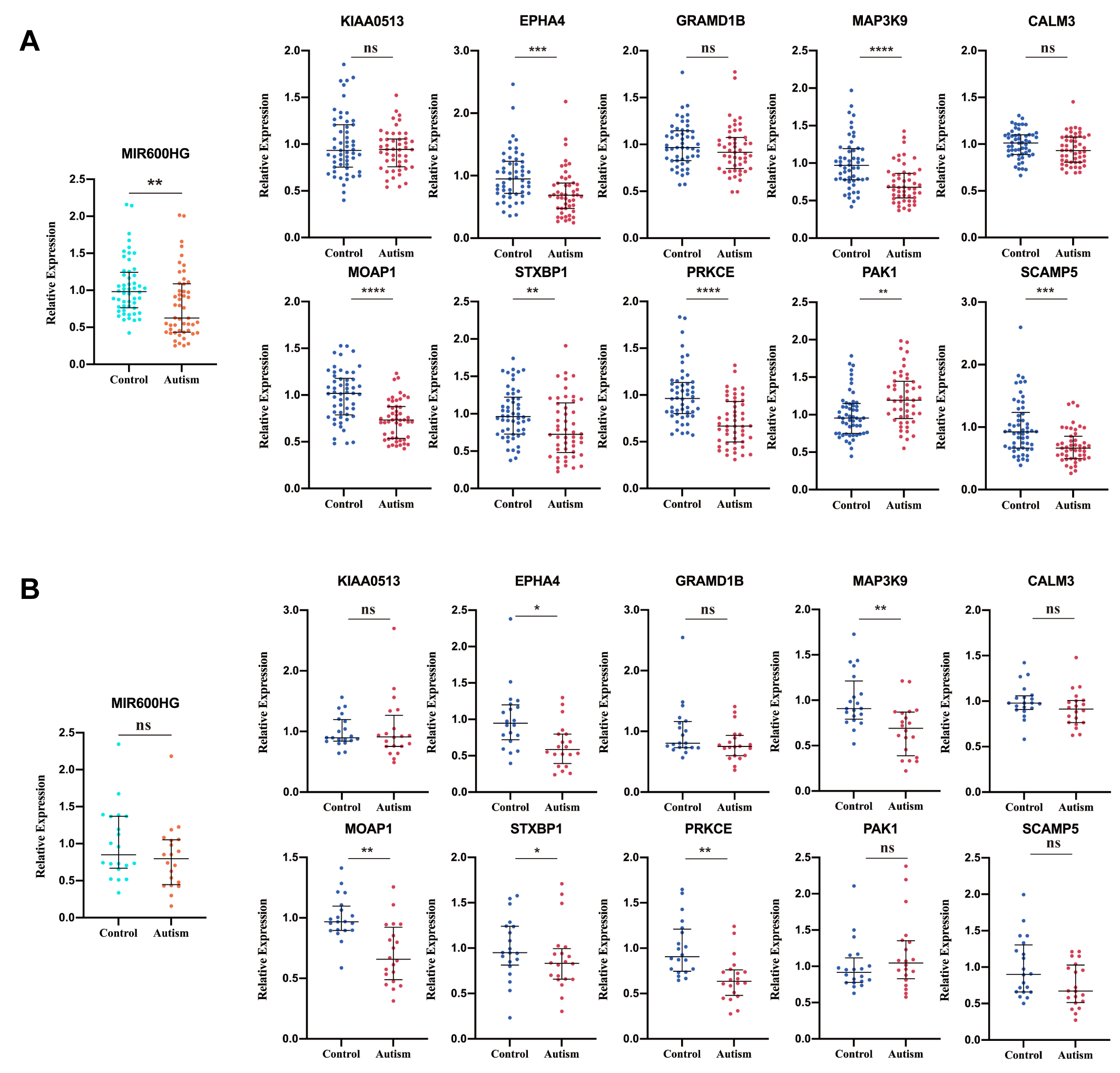


**Supplementary Fig. S5**. Validation of the ceRNA network marker genes in clinical (peripheral blood) samples. (A) Expression of 10 hub DEmRNAs and 1 hub DElncRNA in male patients with autism (n = 50) and healthy controls (n = 55). (B) Expression of 10 hub DEmRNAs and 1 hub DElncRNA in female patients with autism (n = 20) and healthy controls (n = 20). Mann–Whitney U test was used for statistical analysis, and FDR was used for multiple testing corrections. * FDR < 0.05, ** FDR < 0.01, *** FDR < 0.001, **** FDR < 0.0001.


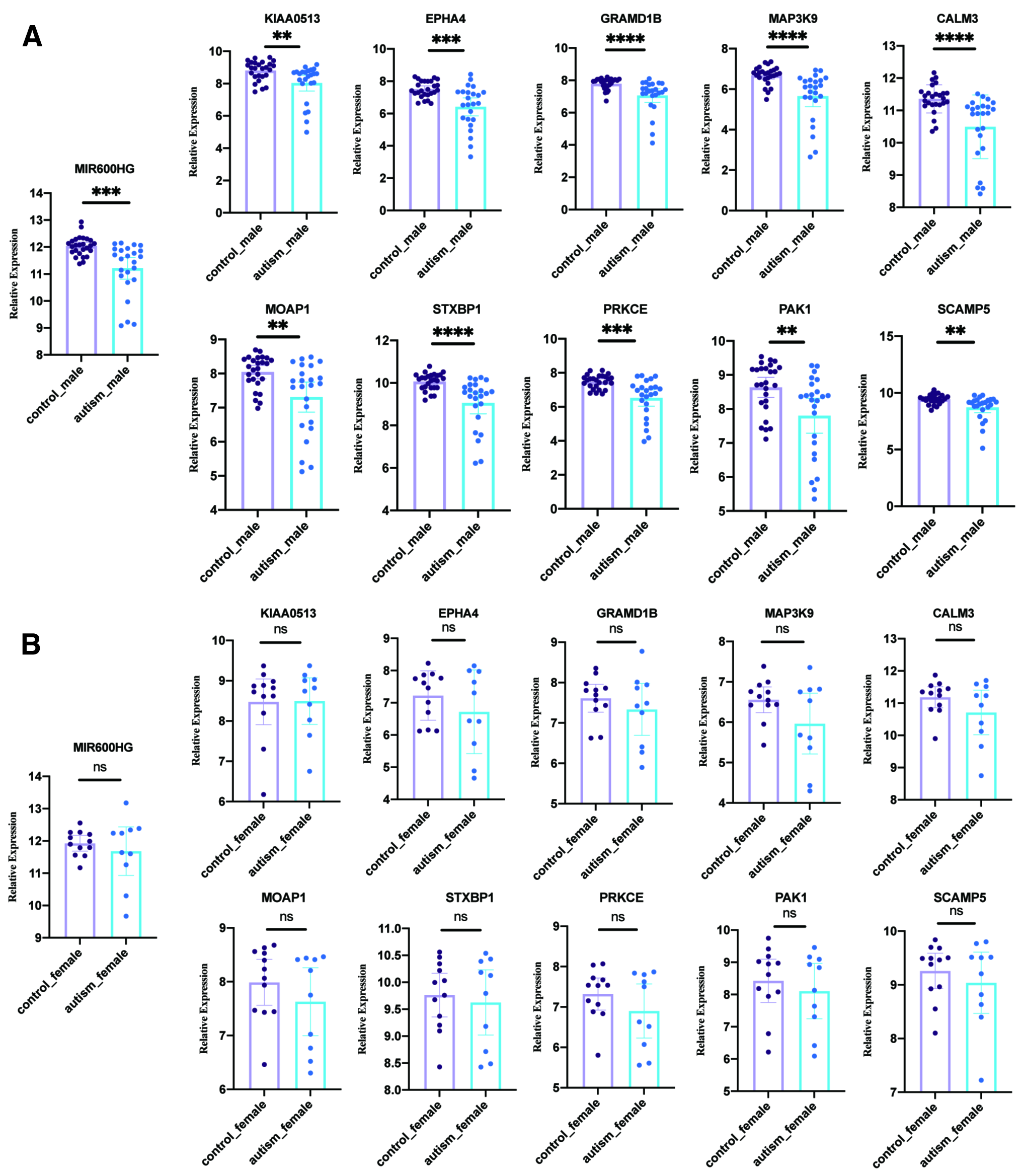


**Supplementary Fig. S6**. The ceRNA network marker genes in GSE59288 (autism patients: n = 34) and GSE51264 (normal samples: n = 38) of prefrontal cortex samples. (A) Expression of 10 hub DEmRNAs and 1 hub DElncRNA in male patients with autism (n = 24) and healthy controls (n = 26). (B) Expression of 10 hub DEmRNAs and 1 hub DElncRNA in female patients with autism (n = 10) and healthy controls (n = 12). Mann–Whitney U test was used for statistical analysis, and FDR was used for multiple testing corrections. * P < 0.05, ** P < 0.01, *** P < 0.001, **** P < 0.0001.


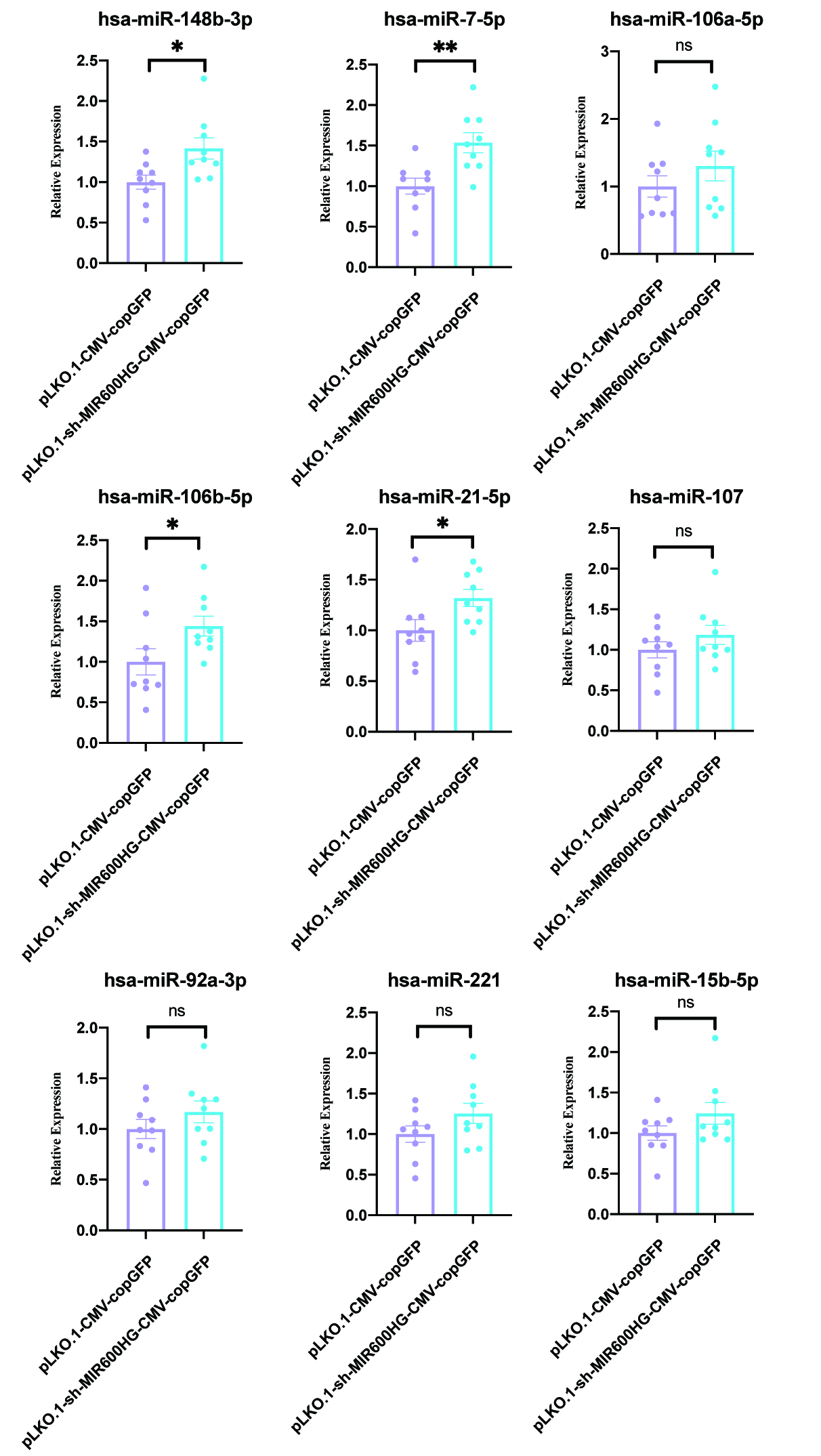


**Supplementary Fig. S7.** Experimental validation of the miRNA expression associated with MIR600HG following shRNA knockdown of this lncRNA. Data are presented as individual data points, with bar plots showing the mean and standard deviation (n = 9 each group). Data represent relative miRNA expression, normalized to U6 snRNA expression (2^−∆∆Ct^). Statistical significance was calculated by Student's t test, * P < 0.05, **P < 0.01, Mean ± SEM.


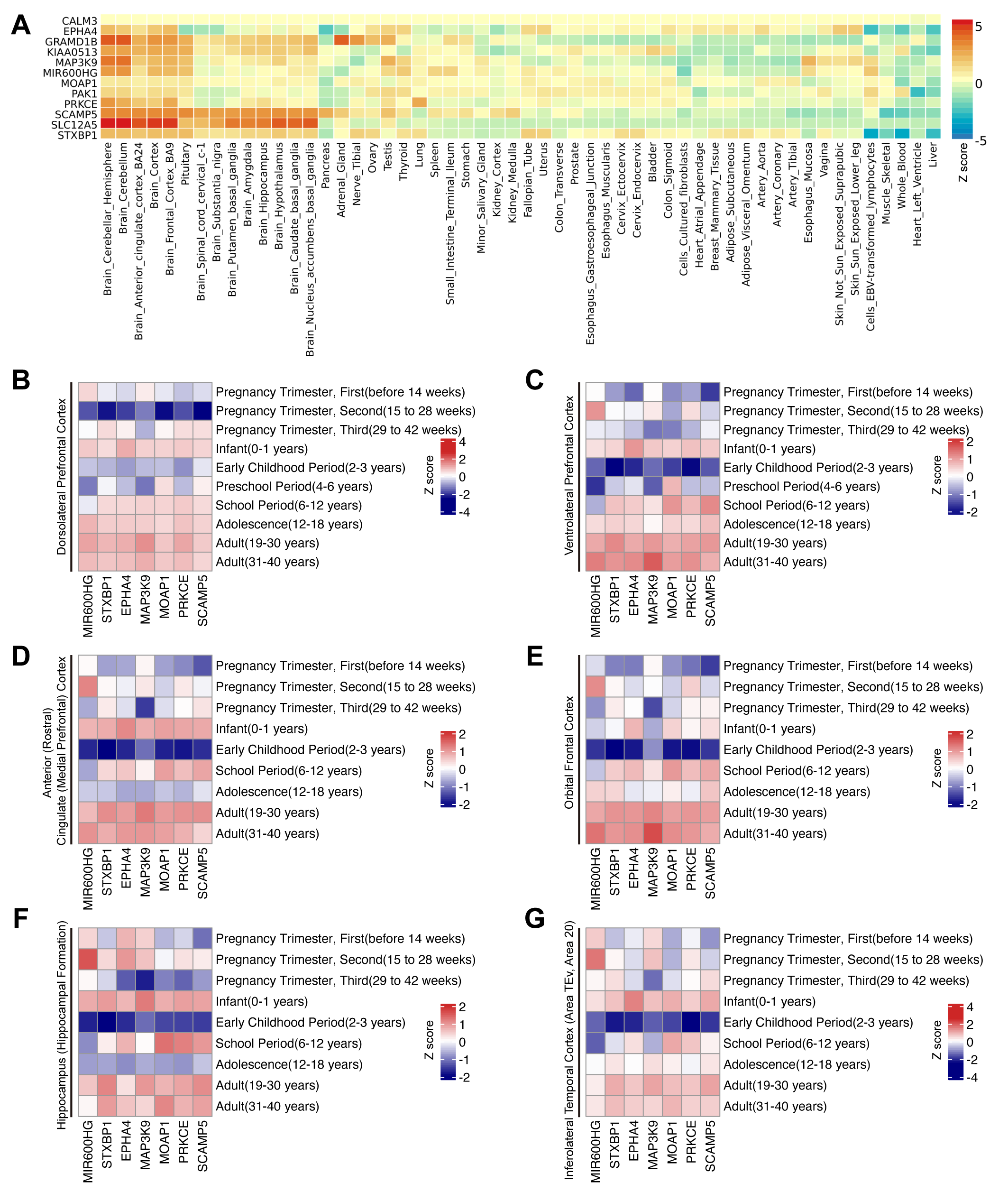


**Supplementary Fig. S8**. Gene expression heatmap. (A) Gene expression heatmap in 54 tissue types from the GTEx dataset. Average normalized expression per label. (B–G) Heatmaps showing the expression patterns of *MIR600HG* and target mRNAs in different human brain regions and at different developmental stages.

**
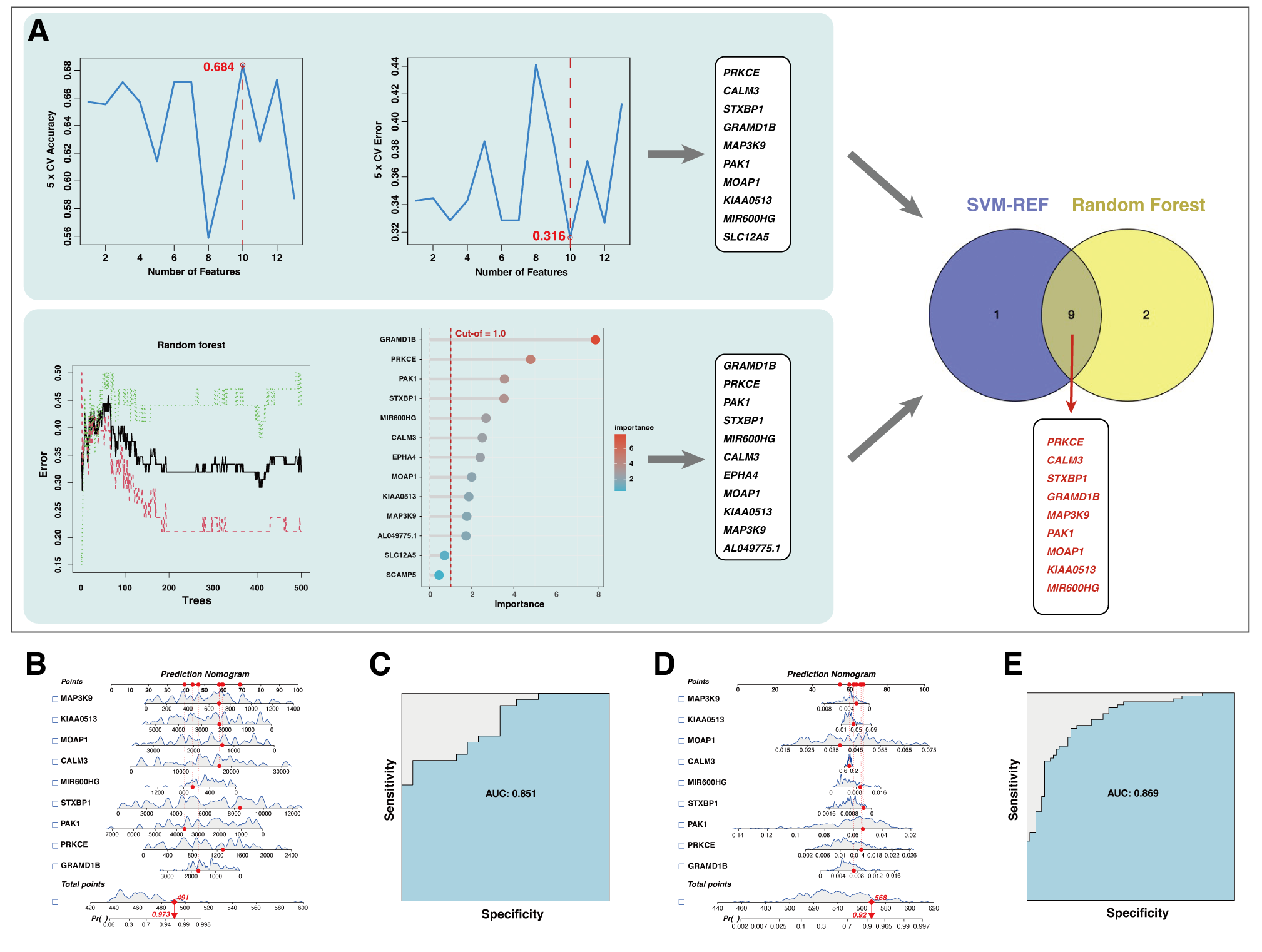
**

**Supplementary Fig. S9**. Machine learning algorithms were applied for diagnostic modeling. (A) SVM-RFE and RF module based on the characteristic gene. The Venn diagram shows the overlapping genes in SVM and RF modules. (B) Nomogram predicting ASD probability in GSE59288 (autism patients: n = 34) and GSE51264 (normal samples: n = 38) of prefrontal cortex samples. (C) The AUC curve for the diagnostic nomogram in prefrontal cortex samples. (D) Nomogram predicting ASD probability in clinical (peripheral blood) samples (autism: control = 70: 75). (E) The AUC curve for the diagnostic nomogram in peripheral blood samples. Abbreviations: AUC, area under the curve; SVM-RFE, support vector machine (SVM) recursive feature elimination; RF, random forest.
